# Changes in Three-Dimensional Volumetric Liver Fat Fraction after Cure of Chronic Hepatitis C Infection With Direct-Acting Antivirals

**DOI:** 10.1093/ofid/ofag023

**Published:** 2026-01-15

**Authors:** Jessie Torgersen, Craig W Newcomb, Dean M Carbonari, Shanae M Smith, Katherine L Brecker, Chamith S Rajapakse, Brandon C Jones, Christiana Cottrell, Rasleen Grewal, Jennifer C Price, Joshua F Baker, Jay R Kostman, Stacey Trooskin, Rebecca A Hubbard, Babette S Zemel, Mary B Leonard, Vincent Lo Re

**Affiliations:** Department of Medicine, Perelman School of Medicine, University of Pennsylvania, Philadelphia, Pennsylvania, USA; Department of Biostatistics, Epidemiology, and Informatics, Center for Clinical Epidemiology and Biostatistics, Center for Real-world Effectiveness and Safety of Therapeutics, Perelman School of Medicine, University of Pennsylvania, Philadelphia, Pennsylvania, USA; Department of Biostatistics, Epidemiology, and Informatics, Center for Clinical Epidemiology and Biostatistics, Center for Real-world Effectiveness and Safety of Therapeutics, Perelman School of Medicine, University of Pennsylvania, Philadelphia, Pennsylvania, USA; Department of Biostatistics, Epidemiology, and Informatics, Center for Clinical Epidemiology and Biostatistics, Center for Real-world Effectiveness and Safety of Therapeutics, Perelman School of Medicine, University of Pennsylvania, Philadelphia, Pennsylvania, USA; Department of Biostatistics, Epidemiology, and Informatics, Center for Clinical Epidemiology and Biostatistics, Center for Real-world Effectiveness and Safety of Therapeutics, Perelman School of Medicine, University of Pennsylvania, Philadelphia, Pennsylvania, USA; Department of Biostatistics, Epidemiology, and Informatics, Center for Clinical Epidemiology and Biostatistics, Center for Real-world Effectiveness and Safety of Therapeutics, Perelman School of Medicine, University of Pennsylvania, Philadelphia, Pennsylvania, USA; Department of Radiology, Perelman School of Medicine, University of Pennsylvania, Philadelphia, Pennsylvania, USA; Department of Orthopedic Surgery, Perelman School of Medicine, University of Pennsylvania, Philadelphia, Pennsylvania, USA; Department of Radiology, Perelman School of Medicine, University of Pennsylvania, Philadelphia, Pennsylvania, USA; Department of Bioengineering, University of Pennsylvania, Philadelphia, Pennsylvania, USA; Department of Radiology, Perelman School of Medicine, University of Pennsylvania, Philadelphia, Pennsylvania, USA; Department of Radiology, Perelman School of Medicine, University of Pennsylvania, Philadelphia, Pennsylvania, USA; Department of Medicine, University of California, SanFrancisco School of Medicine, San Francisco, California, USA; Department of Medicine, Perelman School of Medicine, University of Pennsylvania, Philadelphia, Pennsylvania, USA; Department of Biostatistics, Epidemiology, and Informatics, Center for Clinical Epidemiology and Biostatistics, Center for Real-world Effectiveness and Safety of Therapeutics, Perelman School of Medicine, University of Pennsylvania, Philadelphia, Pennsylvania, USA; Philadelphia FIGHT, Philadelphia, Pennsylvania, USA; Mazzoni Center, Philadelphia, Pennsylvania, USA; Department of Biostatistics, Brown University School of Public Health, Providence, Rhode Island, USA; Department of Pediatrics, Children's Hospital of Philadelphia, Philadelphia, Pennsylvania, USA; Department of Pediatrics, Stanford University School of Medicine, Palo Alto, California, USA; Department of Medicine, Perelman School of Medicine, University of Pennsylvania, Philadelphia, Pennsylvania, USA; Department of Biostatistics, Epidemiology, and Informatics, Center for Clinical Epidemiology and Biostatistics, Center for Real-world Effectiveness and Safety of Therapeutics, Perelman School of Medicine, University of Pennsylvania, Philadelphia, Pennsylvania, USA

**Keywords:** hepatic steatosis, hepatitis C, liver fat fraction, magnetic resonance imaging

## Abstract

**Background:**

It remains unclear if cure of hepatitis C virus (HCV) infection with direct-acting antivirals (DAAs) leads to change in liver fat fraction and if this change differs from people without HCV observed over similar time. We evaluated the change in three-dimensional volumetric liver fat fraction by magnetic resonance imaging (MRI) prior to DAA treatment and 18 months following initiation and compared changes in uninfected controls over 18 months.

**Methods:**

We conducted a cohort study of 35 participants who initiated DAAs and achieved cure and 42 without HCV infection as controls. At enrollment and 18 months later, participants had measurements of height, body weight, and three-dimensional volumetric liver fat fraction and cross-sectional area of abdominal subcutaneous and visceral adipose tissue by MRI. Multivariable linear regression was used to estimate group differences in mean changes in liver fat fraction.

**Results:**

Mean change in three-dimensional volumetric liver fat fraction between Months 0 and 18 was +2.89% (95% CI: +.89%, +4.88%) among participants with cured HCV and +0.48% (95% CI: −1.02%, +1.97%) among controls (between group difference *P*-value = .05). After adjustment for age, sex, smoking, and change in body mass index, the mean difference in liver fat fraction change between Months 0 and 18 was +3.11% (95% CI: +.46%, +5.76%; *P* = .022) higher for participants with HCV versus controls.

**Conclusions:**

Three-dimensional volumetric liver fat fraction by MRI significantly increased between Months 0 and 18 following successful treatment of HCV infection, but this finding was not observed from Month 0 to 18 among participants without HCV infection.

Hepatic steatosis, defined by >5% hepatocyte lipid accumulation, is prevalent among people living with chronic hepatitis C virus (HCV) infection, since both viral and metabolically-mediated factors can promote excess hepatocyte lipid deposition [1, 2]. We recently used a novel three-dimensional volumetric assessment by magnetic resonance imaging (MRI) to show that people living with chronic HCV infection had higher mean volumetric liver fat fraction compared with people without HCV infection, independent of age, sex, and body mass index (BMI) [3]. Unlike liver biopsy, which is subject to sampling bias [4] and indication bias [5], or magnetic resonance spectroscopy, which quantifies fat in limited regions of the liver using a two-dimensional approach, three-dimensional volumetric assessment by MRI allows for evaluation of fat throughout the entire liver [6–8]. Since hepatic steatosis may not be homogeneous in distribution [9], three-dimensional volumetric assessment provides a more comprehensive determination of fat throughout the hepatic parenchyma. Hepatic steatosis in the setting of chronic HCV can accelerate progression of liver fibrosis [10], but the impact of HCV treatment on hepatic steatosis is unclear.

Prior cohort studies conducted during the interferon and early direct-acting antiviral (DAA) era suggested that modest decreases in liver fat may be observed following viral eradication [11–13]. However, these studies were performed primarily among Asian and European patients. It remains unclear if such changes occur among patients in the United States (US) with contemporary DAAs. Moreover, it is unknown if changes in liver fat following cure of HCV infection with DAA therapy differ from those observed over time in people without HCV.

We evaluated changes in liver fat fraction, determined using three-dimensional volumetric assessment by MRI, among people with chronic HCV infection following successful DAA treatment compared with people without HCV infection. We measured liver fat fraction in people with chronic HCV prior to DAA treatment and 18 months later (∼1 year following HCV cure). We compared the change with people without HCV using the same modality at enrollment and 18 months later.

## METHODS

### Study Design and Setting

Details of the study design and setting have been previously published [3]. Briefly, we conducted a prospective cohort study of DAA-naïve people with chronic HCV infection and those without HCV infection. People with chronic HCV infection were recruited from viral hepatitis and primary care practices within the University of Pennsylvania Health System and from Philadelphia FIGHT. Primary care practices at Penn served as recruitment sites for people without HCV infection. Study personnel recruited individuals who presented to their primary care provider for routine care visits to serve as controls. All clinics were located in Philadelphia, Pennsylvania.

Patients scheduled for clinical visits were prescreened for HCV infection and, following approval from their healthcare provider, were approached by study personnel for interest in participation. Self-reported responses were used to evaluate possible eligibility, which was confirmed via medical record review after obtaining informed consent. We used stratified sampling to recruit people without HCV according to sex assigned at birth, self-identified race (Black vs not Black), and age category (18–39, 40–59, ≥60 years) to promote balance in demographic characteristics between cohorts. Participants were recruited from 1 January 2019 through 31 July 2022.

After enrollment, all participants underwent a baseline (Month 0) assessment of demographic and clinical characteristics, height and body weight, serum lipids, and three-dimensional volumetric liver fat fraction and cross-sectional area of abdominal subcutaneous and visceral adipose tissue by MRI. At follow-up (Month 18), assessment of height and body weight, serum lipids, and MRI measurements were repeated. Cure of HCV was defined as the most recent HCV RNA undetectable.

### Patient Consent Statement

All participants provided written informed consent. The study was approved by the Institutional Review Board (IRB) of the University of Pennsylvania, which served as the IRB of Record.

### Study Participants

People with chronic HCV infection were eligible if they were ≥18 years of age, had detectable HCV RNA, and had hepatic fibrosis staging <6 months prior to enrollment. People with chronic HCV who had HIV-1 coinfection were eligible for inclusion if they were on a stable antiretroviral therapy (ART) regimen for ≥4 weeks before enrollment with documented HIV RNA <200 copies/mL. We allowed inclusion of people with HIV in the cohort of participants with HCV because in our prior study evaluating hepatic steatosis using three-dimensional volumetric assessment by MRI, we observed no statistically significant difference in liver fat fraction between people with HIV/HCV coinfection and either those with HCV monoinfection or controls without either viral infection [3].

People without HCV infection were eligible for inclusion as controls if they were ≥18 years of age and did not have HCV or HIV infection. At both the Month 0 and Month 18 study visits, controls were confirmed to be HCV and HIV antibody-negative by OraSure's OraQuick® assay.

Patients were excluded if review of medical records identified or patients self-reported conditions that might affect body composition or liver fat, including chronic kidney disease (estimated glomerular filtration rate <60 mL/min/1.73 m^2^) [14]; cancer (excluding non-melanomatous skin malignancy); malabsorption (due to celiac disease, small bowel resection surgery, chronic diarrhea); acute weight loss >5% of body weight over previous 3 months; or another chronic liver disease (ie, hepatitis B, hemochromatosis, alpha-1-antitrypsin, autoimmune hepatitis, sclerosing cholangitis, biliary cirrhosis, Wilson's disease, metabolic dysfunction-associated steatotic liver disease). People with chronic HCV were excluded if they had: (1) HCV genotype 3 infection (which promotes hepatic steatosis [15, 16]); or (2) previously received DAA-based HCV therapy (since prior HCV treatment might affect hepatic steatosis eradication [11, 12, 17]). We aimed to include 50 participants per group to provide 80% power to detect a difference in Month 0–18 change in liver fat fraction of at least 5%, assuming a type 1 error rate of 0.05% and 5% standard deviation in within-group change in liver fat fraction.

### Assessment of Demographic, Clinical, and Anthropometric Data

Data collected at Month 0 included: age; sex assigned at birth; self-identified race and ethnicity; smoking status; alcohol consumption in the past year determined by the 10-question Alcohol Use Disorders Identification Test [18]; drug use in the past year determined by the 10-question Drug Abuse Screening Test [19]; and history of injection drug use. Data collected from all participants’ medical records within 12 months prior to enrollment included: diabetes mellitus (defined by diagnosis [ever], hemoglobin A1c ≥ 6.5%, or random glucose ≥200 mg/dL) and serum creatinine. Among participants with chronic HCV, we reviewed records to determine HCV diagnosis date, most recent pretreatment HCV RNA and genotype, and pretreatment liver fibrosis stage by vibration-controlled transient elastography or proprietary serologic fibrosis assessment [20].

Body weight and height were measured at Months 0 and 18 using a digital scale (Scaltronix) and stadiometer (Holtain), respectively. Physical activity in a typical week over the prior month was assessed at Months 0 and 18 using the Multi-Ethnic Study of Atherosclerosis questionnaire [21].

### Measurement of Hepatic, Subcutaneous, and Visceral Fat

A 3-Tesla whole-body MR imager (Siemens Prisma; Erlangen, Germany) was used for three-dimensional volumetric assessment of liver fat fraction and determination of the cross-sectional area of abdominal subcutaneous and visceral adipose tissue, as previously described [3]. For computation of liver fat fraction, the liver boundary on all slices was segmented, and the liver fat fraction was computed as the voxel-wise median fat fraction (%) across the entire three-dimensional liver volume. A decrease in liver fat fraction of >30% is considered significant [22].

### Laboratory Data

Non-fasting blood samples were collected for serum total cholesterol, high-density lipoprotein, low-density lipoprotein, and triglyceride levels (Roche Cobas c311; Roche Diagnostics) at Months 0 and 18.

### Statistical Analysis

Differences in characteristics at Month 0 between participants with HCV infection and controls were assessed using Wilcoxon rank-sum tests for continuous variables and chi-square tests for categorical variables. To assess whether cure of HCV with DAA therapy resulted in quantitative change in hepatic steatosis, we assessed the mean change in three-dimensional volumetric liver fat fraction between Months 0 and 18 in the two cohorts. We used multivariable linear regression to estimate the mean difference (95% confidence interval [CI]) in liver fat fraction change between the groups. Potential confounding variables were selected *a priori* based on prior literature and plausible biological mechanisms. The final multivariable models were further informed by differences in baseline characteristics between the participants with HCV infection and controls. Models were adjusted for age (continuous), sex, change in BMI (continuous), and current smoking. We did not adjust for drug use because this was colinear with HCV infection. As a sensitivity analysis, we repeated the primary analysis, replacing BMI change with visceral fat cross-sectional area change. As an additional sensitivity analysis, we repeated the primary analysis, excluding patients with advanced hepatic fibrosis/cirrhosis (METAVIR stage F3–F4).

We conducted two secondary analyses. First, to explore the impact of HCV cure on change in liver fat fraction among people with HIV/HCV coinfection, we repeated the analysis, stratifying the HCV cohort by HIV coinfection status. We used multivariable linear regression to estimate mean differences (95% CI) in Month 0–18 changes in liver fat fraction between the control group and people with: (1) HIV/HCV coinfection, and (2) HCV alone. Second, we used multivariable linear regression to explore the association between baseline hepatic fibrosis stage (F3–F4 vs F0–F2) and change in liver fat fraction from Month 0 to Month 18 among the cohort of people with HCV infection. Analyses were conducted using SAS 9.4 (SAS Institute Inc.).

## RESULTS

### Participant Characteristics

Among 519 patients screened for eligibility, 172 were potentially eligible based on self-report; after medical record review, 64 were excluded (reasons reported in Figure 1). The Month 0 visit was completed by 108 participants (54 with chronic HCV; 54 without HCV). After Month 0, 16 people with chronic HCV and 10 people without HCV withdrew from the study (reasons reported in Figure 1). A total of 38 people with chronic HCV and 41 people without HCV completed Month 0 and Month 18 assessments (Figure 1). Among the 38 people with HCV, 3 (8%) did not achieve cure and were excluded. The final sample included 76 participants (35 people with HCV; 41 controls).

**Figure 1.**
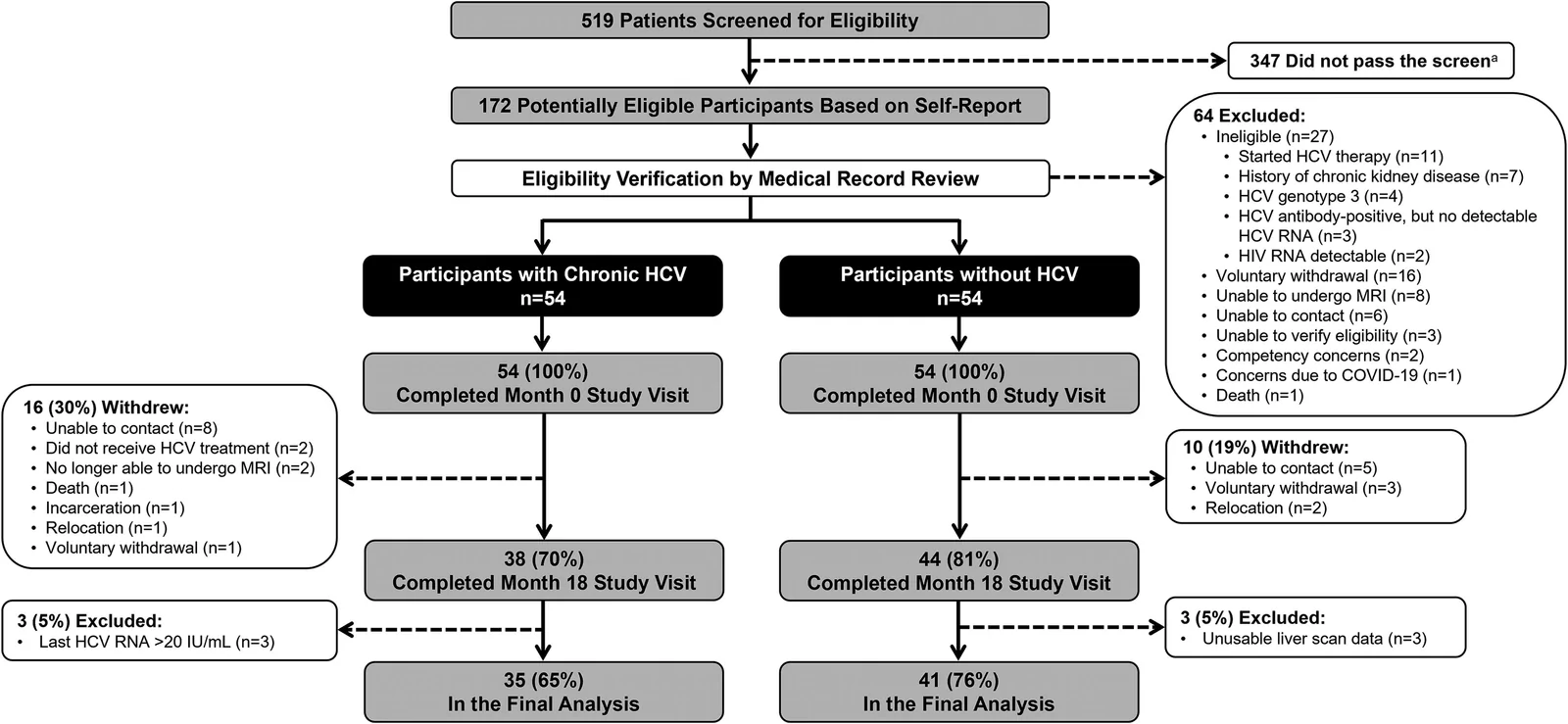
Cohort selection flow. Abbreviations: HCV, hepatitis C virus; HIV, human immunodeficiency virus; RNA, ribonucleic acid. ^a^Screening questions have been previously published Torgersen J, et al. PLoS One 2025. DOI:10.1371/journal.pone.0327668.

Participants with HCV were more likely to be older, male, current smokers, report prior drug use, and have a lower median BMI than participants without HCV (Table 1). No other significant differences in demographic or clinical characteristics between the groups were observed. Eight people with chronic HCV had advanced hepatic fibrosis/cirrhosis. Eleven (31%) people with chronic HCV also had HIV coinfection (median CD4+ cell count = 385 cells/mm^3^) with undetectable HIV RNA on stable ART (10 received a regimen containing tenofovir alafenamide; 1 received a regimen with dolutegravir/abacavir/lamivudine).

**Table 1.** Baseline Characteristics of Participants, by Chronic Hepatitis C Virus Infection Status

Characteristic	Participants With Chronic HCV (*n* = 35)	Participants Without HCV (*n* = 41)	*P*-Value
Median (IQR) age, years	60 (44–65)	54 (36–61)	**.040**
Missing	0 (0.0%)	0 (0.0%)	-
Male sex	29 (82.9%)	25 (61.0%)	**.036**
Missing	0 (0.0%)	0 (0.0%)	-
Race			.257
Asian	0 (0%)	2 (4.9%)	
Black or African American	25 (71.4%)	24 (58.5%)	
White	9 (25.7%)	13 (31.7%)	
Other or Multiracial	1 (2.9%)	2 (4.9%)	
Missing	0 (0.0%)	0 (0.0%)	-
Hispanic ethnicity	2 (5.7%)	3 (7.3%)	.779
Missing	0 (0.0%)	0 (0.0%)	-
Body mass index			
Median (IQR)	27 (24–30)	29 (25–33)	**.040**
Underweight (<18.50 kg/m^2^)	0 (0.0%)	0 (0.0%)	
Normal (18.50–24.99 kg/m^2^)	12 (34.3%)	10 (24.4%)	
Overweight (25.00–29.99 kg/m^2^)	15 (42.9%)	12 (29.3%)	.103
Obesity (≥30.00 kg/m^2^)	8 (22.9%)	19 (46.3%)	
Missing	0 (0.0%)	0 (0.0%)	-
Median fat mass index (IQR), kg/m^2^	8 (6–9)	9 (6–12)	.110
Missing	0 (0.0%)	0 (0.0%)	-
Metabolic comorbidities			
Diabetes mellitus^a^	10 (28.6%)	8 (19.5%)	.355
Missing	0 (0.0%)	0 (0.0%)	-
Dyslipidemia^b^	13 (37.1%)	16 (39.0%)	.866
Missing	3 (8.6%)	1 (2.4%)	.233
Median total cholesterol (mg/dL, IQR)	156 (140–184)	171 (149–193)	.158
Missing	3 (8.6%)	1 (2.4%)	.233
Median HDL (mg/dL, IQR)	49 (40–60)	52 (47–63)	.232
Missing	3 (8.6%)	1 (2.4%)	.233
Median LDL (mg/dL, IQR)	95 (77–116)	94 (76–120)	>.999
Missing	3 (8.6%)	1 (2.4%)	.233
Median triglycerides (mg/dL, IQR)	106 (81–129)	92 (67–157)	.346
Missing	3 (8.6%)	1 (2.4%)	.233
Median (IQR) serum creatinine, mg/dL^c^	0.95 (0.84–1.07)	0.93 (0.78–1.06)	.890
Missing	1 (2.9%)	12 (29.3%)	**.002**
Smoking status			
Never	4 (11.4%)	23 (56.1%)	
Former	8 (22.9%)	7 (17.1%)	**<.001**
Current	23 (65.7%)	11 (26.8%)	
Missing	0 (0.0%)	0 (0.0%)	-
Median (IQR) pack years of tobacco use^d^	10.0 (5.0–12.3)	5.0 (2.2–16.4)	.279
Missing	0 (0.0%)	0 (0.0%)	-
Hepatic fibrosis stage^e^			
F0–F2 (low)	26 (76.5%)	-	-
F3–F4 (high)	8 (23.5%)	-	-
Time since HCV diagnosis (years)^f^			
<5 y	18 (51.4%)	-	-
5–10 y	8 (22.9%)	-	-
11–20 y	5 (14.3%)	-	-
21–40 y	3 (8.6%)	-	-
>40 y	1 (2.9%)	-	-
Direct-acting antiviral regimen (initial)			
Glecaprevir/pibrentasvir	21 (60.0%)	-	-
Sofosbuvir/velpatasvir	10 (28.6%)	-	-
Sofosbuvir/ledipasvir	4 (11.4%)	-	-
Alcohol use in past year, AUDIT score			
Median (IQR)	2 (0–6)	2 (1–5)	
<8 (low risk)	29 (82.9%)	37 (90.2%)	.883
8–15 (risky/hazardous)	5 (14.3%)	3 (7.3%)	
16–19 (high-risk)	0 (0.0%)	0 (0.0%)	.606
≥20 (highest risk)	1 (2.9%)	1 (2.4%)	
Missing	0 (0.0%)	0 (0.0%)	-
Self-reported history of injection drug use	17 (48.6%)	0 (0%)	**<.001**
Drug use in past year, DAST-10 score			
Median (IQR)	1 (0–6)	0 (0–1)	.050
0 (none)	17 (48.6%)	29 (70.7%)	
1–2 (low)	7 (20.0%)	11 (26.8%)	
3–5 (moderate)	2 (5.7%)	1 (2.4%)	**.012**
6–8 (substantial)	6 (17.1%)	0 (0%)	
9–10 (severe)	3 (8.6%)	0 (0%)	
Missing	0 (0.0%)	0 (0.0%)	-
People with HIV infection	11 (31.4%)	-	-
CD4+ cell count			
Median (IQR)	385 (296–558)	-	-
<200 cells/mm^3^	1 (9.1%)	-	-
Hepatic fibrosis stage^e^			
F0–F2 (low)	9 (81.8%)	-	-
F3–F4 (high)	2 (18.2%)	-	-
Time since HIV diagnosis (years)^g^			
<5 y	3 (27.3%)	-	-
5–10 y	3 (27.3%)	-	-
11–20 y	2 (18.2%)	-	-
21–40 y	3 (27.3%)	-	-
>40 y	0 (0.0%)	-	-

Results are reported as no. (%) unless otherwise noted. Bold indicates *P* < .05.

Abbreviations: AUDIT, Alcohol Use Disorders Identification Test; DAST, Drug Abuse Screen Test; HCV, hepatitis C virus; HDL, high-density lipoprotein; HIV, human immunodeficiency virus; IQR, interquartile range; LDL, low-density lipoprotein.

^a^Diabetes mellitus was defined as the presence of any of the following: (1) diagnoses of diabetes in the medical record, (2) hemoglobin A1c ≥ 6.5%, or (3) random glucose ≥200 mg/dL.

^b^Dyslipidemia was defined as triglycerides ≥150 mg/dL or HDL <40 mg/dL (if male) or <50 mg/dL (if female).

^c^Serum creatinine levels from the prior 12 m, if available, were collected from the medical record.

^d^For current smokers, smoking pack years was calculated as the average number of cigarets smoked per day divided by 20, then multiplied by the number of years the person has smoked.

^e^METAVIR fibrosis stage approximated from vibration-controlled transient elastography or proprietary serologic fibrosis assay. One participant with chronic HCV had a liver fibrosis assessment that was unable to be determined.

^f^Time since HCV diagnosis based on the self-reported year of initial HCV diagnosis.

^g^Time since HIV diagnosis based on the earliest documentation of HIV diagnosis in the medical record.

### Mean Difference in Month 0–18 Change in Liver Fat Fraction

The mean change in liver fat fraction between Months 0 and 18 was +2.89% (95% CI: +.89%, +4.88%) among people with HCV and +0.48% (95% CI: −1.02%, 1.97%) among controls (*P*-value for between group difference = .05; Table 2). No significant changes were observed in subcutaneous or visceral fat cross-sectional area, BMI, physical activity, or serum lipid levels among people with HCV (Table 2). Among the controls, subcutaneous fat cross-sectional area significantly decreased while visceral fat cross-sectional area significantly increased between Months 0 and 18.

**Table 2.** Unadjusted Mean Change in Three-dimensional Volumetric Liver Fat Fraction by MRI, Cross-sectional Area of Abdominal Subcutaneous and Visceral Adipose Tissue by MRI, Body Mass Index, Physical Activity (Determined Using the Multi-Ethnic Study of Atherosclerosis Questionnaire), and Serum Lipid Levels Between Month 0 and Month 18 in Participants With and Without Hepatitis C Virus Infection

Measurement	Participants With HCV (*n* = 35)			Participants Without HCV (*n* = 41)			Between Group Difference (95% CI)	*P*-Value for Diff in Change
	Mean (SD) at Month 0	Mean (SD) at Month 18	Mean (SD) Change	Mean (SD) at Month 0	Mean (SD) at Month 18	Mean (SD) Change		
Volumetric liver fat fraction (%)	14.19 (6.05)	17.07 (6.90)	**+2.89** (**5.81)**	12.00 (4.55)	12.47 (3.98)	+0.48 (4.74)	+2.41 (−.003, +4.82)	.**050**
Missing, *n* (%)	0 (0.0%)			0 (0.0%)				
SubQ fat cross-sectional area (cm^2^)	327.60 (175.58)	336.79 (192.28)	+9.19 (89.56)	383.54 (215.12)	347.56 (201.74)	**−35.98** (**85.30)**	+45.17 (+4.16, +86.18)	.**031**
Missing, *n* (%)	3 (8.6%)			0 (0%)				
Visceral fat cross-sectional area (cm^2^)	118.77 (55.92)	125.58 (70.95)	+6.81 (42.15)	143.02 (108.65)	160.64 (99.45)	+**17.63** (**47.70)**	−10.82 (−32.15, +10.52)	.316
Missing, *n* (%)	3 (8.6%)			0 (0%)				
Body mass index (kg/m^2^)	27.63 (5.50)	28.04 (6.18)	+0.41 (2.98)	29.44 (6.79)	29.36 (6.44)	−0.08 (1.57)	+0.480 (−.59, +1.55)	.373
Missing, *n* (%)	0 (0%)			0 (0%)				
Physical activity (MET-hours/week)	2.74 (1.38)	2.86 (1.46)	+0.11 (2.00)	4.46 (2.07)	4.29 (1.83)	−0.17 (1.64)	+0.29 (−.55, +1.12)	.497
Missing, *n* (%)	0 (0.0%)			0 (0%)				
Total cholesterol (mg/dL)	162.97 (33.31)	163.67 (42.25)	+0.70 (36.51)	173.10 (33.97)	173.10 (38.50)	0.00 (26.12)	0.70 (−14.35, +15.75)	.926
Missing, *n* (%)	5 (14.3%)			2 (4.9%)				
High-density lipoprotein (mg/dL)	51.87 (17.10)	46.73 (13.67)	−5.13 (14.88)	55.64 (13.87)	55.15 (14.94)	−0.49 (12.47)	−4.65 (−11.22, +1.93)	.163
Missing, *n* (%)	5 (14.3%)			2 (4.9%)				
Low-density lipoprotein (mg/dL)	96.07 (31.23)	99.43 (36.94)	+3.37 (32.83)	97.97 (31.49)	95.31 (31.07)	−2.67 (21.73)	+6.03 (−7.10, +19.17)	.363
Missing, *n* (%)	5 (14.3%)			2 (4.9%)				
Triglycerides (mg/dL)	118.17 (58.62)	130.27 (81.28)	+12.10 (90.36)	116.80 (71.96)	121.87 (92.40)	+5.08 (54.51)	+7.02 (−28.00, +42.04)	.690
Missing, *n* (%)	5 (14.3%)			2 (4.9%)				

Results are reported as mean (SD) unless otherwise noted. Bold indicates *P* < .05.

Abbreviations: CI, confidence interval; Diff, difference; HCV, hepatitis C virus; MET, metabolic equivalent of task; SD, standard deviation; subQ, subcutaneous.

After adjustment for age, sex, change in BMI, and smoking, the mean difference in liver fat fraction change between Months 0 and 18 was +3.11% (95% CI: +.46%, +5.76%; *P* = .022) higher for people with HCV versus controls (Table 3). After BMI was replaced with visceral fat cross-sectional area (Table 3), the mean difference in liver fat fraction change remained higher for people with HCV (+3.44%; 95% CI: +.80%, +6.08%; *P* = .012). When we repeated the primary analysis excluding the 8 participants with advanced hepatic fibrosis/cirrhosis (METAVIR stage F3–F4), the adjusted mean difference in liver fat fraction change was higher for people with HCV (+2.21%; 95% CI: −.65%, +5.06%), but this difference was not statistically significant (*P* = .127; Supplementary Table 1).

**Table 3.** Adjusted Mean Differences (95% Confidence Intervals) in the Change in Three-dimensional Volumetric Liver Fat Fraction From Month 0 to Month 18 Between Participants With and Without Chronic Hepatitis C Virus Infection, Overall and Stratified by HIV Status

Model Adjustment	HIV/HCV & HCV Combined (*n* = 35)		HIV/HCV Only (*n* = 11)		HCV Only (*n* = 24)	
	Mean Difference in LFF Change versus Controls^a^ (95% CI)	*P*-Value	Mean Difference in LFF Change versus Controls^a^ (95% CI)	*P*-Value	Mean Difference in LFF Change versus Controls^a^ (95% CI)	*P*-Value
Age, sex, current smoking, change in body mass index	+3.11% (+.46%, +5.76%)	**.022**	+4.18% (+.69%, +7.67%)	.**020**	+2.39% (−.68%, +5.46%)	.125
Age, sex, current smoking, change in visceral fat cross-sectional area	+3.44% (+.80%, +6.08%)	**.012**	+4.39% (+.92%, +7.87%)	.**014**	+2.78% (−.29%, +5.85%)	.075

Bold indicates *P* < .05.

Abbreviations: CI, confidence interval; HCV, hepatitis C virus; LFF, liver fat fraction.

^a^Controls (*n* = 41) included people without both HCV and HIV infection.

When we stratified the cured HCV group by HIV status and repeated the primary adjusted analysis (Table 3), the mean difference in Month 0–18 change in liver fat fraction was +4.18% (95% CI: +.69%, +7.67%; *P* = .020) higher for people with cured HCV plus HIV infection versus controls. The mean difference in Month 0–18 change in liver fat fraction was +2.39% (95% CI: −.68%, +5.46%; *P* = .125) higher for people with cured HCV and no HIV compared with controls.

When examining the association between baseline hepatic fibrosis stage and change in liver fat fraction following cure among the cohort with HCV infection, the adjusted mean change in liver fat fraction was +2.82% (95% CI: −2.49%, +8.12%) higher for those with baseline advanced hepatic fibrosis/cirrhosis (METAVIR F3-F4) than for those without (METAVIR F0-F2), but this difference was not statistically significant (*P* = .286; Supplementary Table 2).

## DISCUSSION

We found that three-dimensional volumetric liver fat fraction measured by MRI significantly increased between Months 0 and 18 following successful treatment of HCV infection, but this finding was not observed from Month 0 to 18 among participants without HCV infection. This increase in liver fat fraction among people with HCV was not accompanied by significant changes in BMI, abdominal subcutaneous or visceral fat, physical activity, or lipid profiles. After controlling for age, sex, and BMI change, the mean Month 0–18 change in liver fat fraction remained significantly larger for participants with HCV compared with controls. This association remained in sensitivity analyses replacing BMI with visceral fat cross-sectional area. While the observed increase in liver fat fraction was modest (+2.89%), this finding suggests that patients cured of their HCV infection may still face metabolic threats to their liver health. Moreover, the mean difference in Month 0–18 change in liver fat fraction was observed to be significantly higher for people with cured HCV plus HIV infection compared with controls.

Few studies have evaluated changes in hepatic fat content following DAA therapy, and results have been conflicting [11–13]. One cohort study of 70 people with chronic HCV from Japan found observed that the median controlled attenuation parameter level decreased significantly following HCV cure from 273 dB/m to 265 dB/m (*P* = .034) [11]. A second cohort study of 198 people with genotype 1 or 2 HCV infection from Japan observed a 0.5% decrease in liver fat fraction, as measured by MRI-proton density fat fraction, 24 weeks after DAA completion [12]. While statistically significant, the magnitude of change was within the expected variation across repeated MRI-proton density fat fraction measurements [23]. A third cohort study of 108 patients from Canada and Italy evaluated liver fat changes, defined by controlled attenuation parameter, between DAA initiation and 24 weeks after completion [13]. The authors observed that the prevalence of hepatic steatosis increased from 47% to 61% following HCV cure. We similarly observed that mean three-dimensional volumetric liver fat fraction by MRI increased following completion of DAA treatment. These findings suggest that liver fat fraction may increase despite HCV cure, and this could contribute to liver fibrosis progression and increase risk of cirrhosis. If our findings are confirmed, future research should evaluate the determinants of increased liver fat fraction following HCV cure to identify potentially modifiable factors that could be intervened upon to prevent accumulation of liver fat.

The reasons why three-dimensional volumetric liver fat fraction significantly increased between Months 0 and 18 following HCV cure relative to controls are unclear. It is possible that eradication of HCV infection with DAA therapy results in improvement in well-being or increases in appetite-inducing hormones, which could result in increased nutritional intake. While mean BMI did not increase significantly among people with HCV infection in this study, weight gain has been observed following successful DAA treatment, even among those who are overweight or obese prior to treatment [24]. Second, our inclusion of people with controlled HIV infection on ART in the cohort of people with HCV infection might have contributed to the increase in liver fat fraction in this group. When evaluating results stratified by HIV status, we did observe that people with cured HCV and HIV had larger gains in liver fat fraction versus controls than did the people with cured HCV but no HIV. HIV can promote hepatic steatosis directly by inducing inflammatory cytokine release through infection of hepatic stellate cells and by enhancing gastrointestinal tract microbial translocation [25, 26]. Use of ART can also promote liver fat deposition by promoting dyslipidemia and insulin resistance [27]. However, these results should be interpreted with caution given the small sample of people with HIV coinfection in the HCV cohort (*n* = 11). Future studies evaluating changes in liver fat fraction following HCV cure should account for these considerations.

Among participants with HCV infection, we observed that the adjusted mean change in volumetric liver fat fraction was higher for those with baseline advanced hepatic fibrosis/cirrhosis (METAVIR F3–F4) compared with those without (METAVIR F0–F2), but the difference in the change was not statistically significant. However, the sample of participants with advanced hepatic fibrosis/cirrhosis was small in this study, and we did not examine liver fibrosis following HCV cure. Future studies should evaluate the influence of baseline hepatic fibrosis and alteration in liver fibrosis stage on changes in liver fat fraction.

We observed that the mean Month 0–18 change in visceral fat cross-sectional area increased in both people with HCV infection and controls. The mean month 0–18 liver fat fraction also increased both among people with HCV infection and controls, though the within-group change was only statistically significant for people with HCV. Notably, the mean Month 0–18 change in subcutaneous fat cross-sectional area significantly decreased among the controls (mean change, −35.98 cm^2^), but not among those with HCV, in whom there was a non-significant increase in subcutaneous fat cross-sectional area (+9.19 cm^2^). It is possible that the significant loss of subcutaneous fat among the controls might have attenuated the increase in visceral fat in this group.

Our study had several limitations. First, we aimed to include 100 participants (50 per group), but we experienced larger than anticipated withdrawals from each cohort due to the reasons specified in Figure 1. Although we adjusted for variables that were imbalanced between the groups (ie, age, sex, change in BMI, baseline smoking), given the small sample size, differences in these variables might still have contributed to some of the study's findings. Moreover, the small sample size of participants with advanced hepatitis fibrosis/cirrhosis could have limited power to detect differences between the groups classified by baseline hepatic fibrosis stage. Second, our study included participants who were mostly middle-aged, Black/African-American, with overweight BMI, which might limit generalizability beyond these groups. Third, we did not collect information on several factors that might have influenced deposition of liver fat, including use of sodium-glucose cotransporter 2 inhibitors or glucagon-like peptide-1 receptor agonists (since the effect of these medications on hepatic steatosis was not fully understood when the study was initiated), diet/food intake, or some comorbidities (eg, thyroid disease, depression). We also did not determine the trajectory of liver fat fraction prior to DAA treatment, which could have been valuable to compare with the trajectory following DAA cure. Fourth, the control group selected for this study might have affected the study conclusions. Because of unexpected study participant withdrawals within each group, the cohort of control participants who completed the study were younger, more commonly female, had higher median BMI, less commonly reported smoking, and less frequently used illicit drugs. Further, our study design did not permit enrollment of control participants with HIV infection. These factors might have contributed to observed changes in liver fat fraction over time among control participants that could have affected group differences. Finally, our study did not evaluate changes in hepatic fibrosis after DAA treatment, so we were unable to determine if the increase in liver fat fraction was associated with change in hepatic fibrosis.

In conclusion, this study observed an increase in mean volumetric liver fat fraction among participants with HCV infection from DAA pretreatment through 18 months after DAA initiation. This increase in liver fat fraction was not associated with changes in BMI or visceral fat cross-sectional area. A similar increase in liver fat fraction over time was not observed in controls without HCV. Further research is needed to identify optimal strategies to improve metabolic health after HCV cure.

## Supplementary Material

ofag023_Supplementary_Data

## Supplementary Data

Supplementary materials are available at *Open Forum Infectious Diseases* online. Consisting of data provided by the authors to benefit the reader, the posted materials are not copyedited and are the sole responsibility of the authors, so questions or comments should be addressed to the corresponding author.
