# Supplementary material for: Changes in Three-Dimensional Volumetric Liver Fat Fraction after Cure of Chronic Hepatitis C Infection With Direct-Acting Antivirals
Source: Open Forum Infect Dis. 2026 Jan 15;13(10):ofag023. doi: 10.1093/ofid/ofag023 (PMC13625188; doi:10.1093/ofid/ofag023)
Supplement: Supplementary file 1 [file ofag023_supplementary_data.docx]

**Changes in Three-Dimensional Volumetric Liver Fat Fraction after Treatment of Chronic Hepatitis C Infection with Direct-Acting Antivirals**

Jessie Torgersen; Craig W. Newcomb; Dean M. Carbonari; Shanae M. Smith; Katherine L. Brecker; Chamith S. Rajapakse; Brandon C. Jones; Christiana Cottrell; Rasleen Grewal; Jennifer C. Price; Joshua F. Baker; Jay R. Kostman; Stacey Trooskin; Rebecca A. Hubbard; Babette S. Zemel; Mary B. Leonard; Vincent Lo Re III

**Supplemental Table 1**……………………………………………………………………….……….2

**Supplemental Table 2**………………………………………………………………….…………….3

**Supplemental Table 1.** Unadjusted and adjusted mean differences (95% confidence interval) in the change in three-dimensional volumetric liver fat fraction from Month 0 to Month 18 between participants with and without HCV infection, after excluding participants with HCV who had advanced hepatic fibrosis/cirrhosis (METAVIR stages F3-F4). Each model is adjusted for the variables indicated.

|  | **Mean Difference (95% CI) in Volumetric Liver Fat Fraction Change (%)** | | | | | | | |
| --- | --- | --- | --- | --- | --- | --- | --- | --- |
|  | **Model #1** | | **Model #2** | | **Model #3** | | **Model #4** | |
| **Characteristics** | **Unadjusted Estimate** | ***P-Value*** | **Adjusted**  **Estimate** | ***P- Value*** | **Adjusted**  **Estimate** | ***P-Value*** | **Adjusted**  **Estimate** | ***P-Value*** |
| Hepatitis C infection | +1.72% (-0.97%, +4.41%) | 0.206 | +2.81% (0.15%, 5.47%) | 0.039 | +2.21% (-0.65%, +5.06%) | 0.127 | +2.52% (-0.33%, +5.37%) | 0.083 |
| Male sex | - |  | -3.24% (-6.06%, -0.42%) | 0.025 | -3.22% (-6.01%, -0.44%) | 0.024 | -2.72% (-5.56%, +0.13%) | 0.061 |
| Age (per 10 years) | - |  | -0.10% (-0.18%, -0.01%) | 0.032 | -0.10% (-0.18%, -0.01%) | 0.030 | -0.10% (-0.19%, -0.01%) | 0.025 |
| Current smoking | - |  | - |  | +0.69% (-2.00%, +3.39%) | 0.610 | +0.27% (-2.45%, +2.98%) | 0.844 |
| Change in body mass index, kg/m^2^ (continuous) | - |  | - |  | +0.49% (-0.03%, +1.00%) | 0.064 | - |  |
| Change in visceral fat cross-sectional area, cm^2^ (continuous) | - |  | - |  | - |  | +0.03% (+0.003%, +0.06%) | 0.031 |

Abbreviations: CI=confidence interval

# **Supplemental Table 2.** Association between baseline hepatic fibrosis stage (METAVIR stage F3-F4 versus F0-F2) and change in three-dimensional volumetric liver fat fraction from Month 0 to Month 18 among the cohort of participants with hepatitis C virus infection. Each model is adjusted for the variables indicated.

|  | **Mean Difference (95% CI) in Volumetric Liver Fat Fraction Change (%)** | | | | | | | |
| --- | --- | --- | --- | --- | --- | --- | --- | --- |
|  | **Model #1** | | **Model #2** | | **Model #3** | | **Model #4** | |
| **Characteristics** | **Unadjusted Estimate** | ***P-Value*** | **Adjusted**  **Estimate** | ***P- Value*** | **Adjusted**  **Estimate** | ***P-Value*** | **Adjusted**  **Estimate** | ***P-Value*** |
| Baseline METAVIR F3-4 vs. F0-F2 | +3.34% (-1.42%, +8.10%) | 0.162 | +3.14% (-2.08%, +8.37%) | 0.229 | +2.82% (-2.49%, +8.12%) | 0.286 | +3.16% (-1.83%, +8.14%) | 0.204 |
| Male sex | - |  | -4.08% (-9.37%, +1.22%) | 0.127 | -4.67% (-10.00%, +0.66%) | 0.083 | -5.21% (-10.18%, -0.24%) | 0.041 |
| Age (per 10 years) | - |  | -0.02% (-0.20%, +0.17%) | 0.855 | +0.02% (-0.18%, +0.21%) | 0.850 | -0.02% (-0.21%, +0.16%) | 0.814 |
| Current smoking | - |  | - |  | +3.33% (-1.05%, +7.71%) | 0.131 | +2.91% (-1.29%, +7.10%) | 0.166 |
| Change in body mass index, kg/m^2^ (continuous) | - |  | - |  | +0.06% (-0.63%, +0.75%) | 0.854 | - |  |
| Change in visceral fat cross-sectional area, cm^2^ (continuous) | - |  | - |  | - |  | +0.06% (+0.01%, +0.11%) | 0.017 |

Abbreviations: CI=confidence interval
